# AI-assisted direct anterior approach *versus* posterolateral approach in total hip arthroplasty: a retrospective cohort study based on artifact-reduced CT 3D reconstruction

**DOI:** 10.3389/fbioe.2025.1509200

**Published:** 2025-04-14

**Authors:** ShanBin Zheng, JiaQing Zhu, ZhiYuan Chen, Xun Cao, TianWei Xia, Chao Zhang, Ji Rong Shen

**Affiliations:** Department of Orthopedics and Traumatology, Affiliated Hospital of Nanjing University of Traditional Chinese Medicine, Nanjing, China

**Keywords:** total hip arthroplasty, combined anteversion angle, femoral anteversion angle, acetabular anteversion angle, direct anterior approach(DAA), posterolateral approach (PLA), three-dimensional reconstruction

## Abstract

**Objective:**

To compare the accuracy of implant positioning and early functional recovery between direct anterior approach (DAA) and posterolateral approach (PLA) in total hip arthroplasty (THA) guided by an artificial intelligence preoperative planning system (AIHIP).

**Methods:**

The study population consisted of 206 patients who underwent DAA surgery and 81 patients who underwent PLA surgery, all of whom were designed preoperatively using AI-HIP, and postoperatively using artefact-reduced CT reconstruction for prosthesis mounting angle measurements and follow-up such as postoperative outcomes. The main assessments included prosthesis positioning accuracy (compared to the preoperative plan): acetabular anterior inclination (AA), femur anterior inclination (FNA), combined anterior inclination (CA), alignment of femoral stem prosthesis and femur; clinical outcomes: operative time, hospital stay, and time to grounding; functional scores: Harris Hip Score, WOMAC, and VAS Pain Score; and biomarkers: haemoglobin, CRP, and IL-6, among others.

**Results:**

All 287 patients completed ≥6-month follow-up. While preoperative femoral/acetabular anteversion showed no intergroup differences (p > 0.05), the direct anterior approach (DAA) demonstrated superior postoperative acetabular anteversion control (20.93 ± 7.54° vs. 24.34 ± 7.93°, p < 0.001) despite comparable femoral anteversion (12.97 ± 6.93° vs. 14.56 ± 7.21°, p = 0.009). AI-assisted predictions exhibited smaller deviations in DAA for both parameters (FNA: 3.12 ± 5.88° vs. 5.59 ± 8.21°, p = 0.005; AA: 0.93 ± 7.54° vs. −4.34 ± 7.93°, p < 0.001). No significant differences emerged in combined anteversion, acetabular abduction, or femoral stem alignment parameters (all p > 0.05). DAA achieved shorter incisions (10.64 ± 0.94 vs. 15.21 ± 1.33 cm, p < 0.001) and hospital stays (7.59 ± 4.18 vs. 9.09 ± 3.65 days, p < 0.001) despite longer operative times (118.67 ± 26.95 vs. 53.27 ± 58.71 min, p < 0.001). Functional recovery favored DAA, with better VAS/Harris scores at 3 months and WOMAC scores at 1 month (all p < 0.05). No intergroup differences were observed in postoperative CK, CRP, Hb, or IL-6 levels (p > 0.05).

**Conclusion:**

Both DAA and PLA approaches resulted in satisfactory postoperative outcomes; however, the DAA approach demonstrated enhanced early postoperative efficacy indicators, as well as improved femoral neck and acetabular anteversion compared to the PLA approach. This study advocates for the preferential adoption of the DAA technique for THA, while also emphasizing the importance of considering individual patient factors, as well as the surgeon’s preferences and expertise.

## 1 Introduction

Total Hip Arthroplasty (THA) is widely recognized as the most definitive and effective intervention for individuals diagnosed with Osteonecrosis of the Femoral Head (ONFH) (Filippo et al., 2021), osteoarthritis of the hip joint (OA) (Günther et al., 2021; Klaus Peter et al., 2021; Manon et al., 2021), Developmental Dysplasia of the Hip (DDH) (Zeng et al., 2017), and Fractures of the neck of the femur in the elderly (FNF) (Bhandari et al., 2019). The surgical techniques commonly employed in THA include the Posterolateral Approach (PLA), Direct Anterior Approach (DAA), among others (Lei et al., 2023). However, there exists considerable debate regarding the optimal surgical approach that yields the most favorable outcomes (Larry et al., 2018). The PLA is frequently favored due to its capacity to provide sufficient exposure for a diverse array of complex hip replacements, coupled with relatively straightforward surgical manipulation and a shorter learning curve, which has contributed to its status as the earliest and most prevalent approach in THA (Higgins et al., 2015). Nonetheless, this technique necessitates the disruption of the external rotator muscle group and the posterior joint capsule, often resulting in the requirement for early postoperative hip restrictions.

Conversely, the DAA employs muscle and neurovascular interspaces to access the hip joint through the muscular interval, thereby preserving the integrity of the posterior soft tissues. This approach is posited to offer several theoretical advantages, including reduced surgical trauma, diminished postoperative pain, and expedited recovery. Consequently, an increasing number of surgeons are adopting the DAA for THA (Dragan et al., 2018; Chechik et al., 2013), with data indicating that approximately half of the members of the American Association of Hip and Knee Surgeons (AAHKS) utilize this technique (Matthew et al., 2023). However, the DAA is not without its limitations, particularly concerning its relative lack of exposure and the challenges associated with elevating the proximal femur during the procedure, which may lead to suboptimal positioning of the femoral stem prosthesis.

The ongoing discourse regarding the DAA’s potential benefits in terms of postoperative neck anteversion, alignment of the femoral stem, early pain alleviation, and functional recovery remains significant (Fatih et al., 2019; Jia et al., 2019; Hirohito et al., 2015). Ultimately, the selection of surgical approach is predominantly influenced by the surgeon’s individual preferences (Meermans et al., 2017). Accurate alignment of the femoral stem, appropriate positioning of the acetabular cup, and correct anteversion angles of both the acetabular and femoral stems are essential for mitigating complications such as prosthesis dislocation, wear, and component loosening, thereby enhancing the overall quality of life for patients (Hirohito et al., 2015). Misalignment of the femoral stem can precipitate complications associated with hip impingement, including dislocation of the prosthesis, accelerated wear and failure of the liner, and loosening of the prosthesis. This risk is particularly pronounced in patients exhibiting hip varus, prominence of the iliac wing, and significant muscular strength, which may contribute to unsatisfactory alignment following femoral stem implantation. Improved alignment of the prosthesis not only significantly decreases the likelihood of related complications but also enhances the mobility of the patient’s joint (Hirohito et al., 2015).

The artificial intelligence preoperative planning system (AI-HIP) has exhibited remarkable reliability in forecasting component sizes, acetabular anteversion, and femoral anteversion for primary THA (Purbasari et al., 2023; Xuzhuang et al., 2021). Research conducted by Huo et al.reveals (Huo et al., 2021) that the AI-HIP system significantly outperforms Mimics software and radiographic assessments in accurately predicting acetabular and femoral stem prosthesis models. Furthermore, Ding et al. reported (Xuzhuang et al., 2021) that the accuracy rates for predicting acetabular cup prosthesis models using AI-HIP and X-ray template measurements were 87.7% and 58.9%, respectively, while the corresponding rates for femoral stem prosthesis models were 94.0% and 65.2%. These results indicate that AI-based systems, such as AI-HIP, demonstrate a high level of precision in preoperative planning for THA. In the present study, all THA procedures employed the AI-HIP system for the preoperative planning of prosthesis models, as well as for determining acetabular and femoral stem anteversion angles. It is posited that this approach contributes positively to reducing the incidence of intraoperative surgical manipulations and minimizing surgical trauma. In this study, we used the AI-HIP system for preoperative planning and compared AI-HIP preoperative planning with postoperative CT 3D reconstruction measurements to assess the impact of direct anterior approach (DAA) and posterolateral approach (PLA) on the positioning accuracy of prosthetic components. With this approach, combined with surgical efficiency, functional outcome, and biological metrics, we comprehensively assessed the differences between the two different approaches, DAA and PLA, in THA surgery.

## 2 Clinical data

### 2.1 Materials and methods

#### 2.1.1 Criteria for inclusion and exclusion of cases

This research received approval from the Ethics Committee of Jiangsu Hospital of Traditional Chinese Medicine and was registered with the China Clinical Trial Center. The study adhered to the principles outlined in the Declaration of Helsinki and followed pertinent guidelines. Prior to participation, written informed consent was obtained from all subjects. Between May 2022 and July 2024, patients undergoing their primary THA at our institution were evaluated based on the following inclusion criteria: (1) Patients undergoing their first THA; (2) Individuals diagnosed with conditions such as developmental dysplasia of the hip, avascular necrosis of the femoral head (attributable to alcoholic, hormonal, traumatic, or idiopathic factors), femoral neck fractures, hip joint osteoarthritis, and other conditions with definitive surgical indications necessitating THA; (3) Preoperative and postoperative evaluations were performed, including a combined CT scan of the hip and knee. The preoperative scans were uploaded to the AI-HIP system for analysis and the postoperative scans were used to measure relevant angles and component alignment. In addition, reliable follow-up data was provided to ensure a comprehensive assessment. (4) Utilization of the AI-HIP system for preoperative planning; (5) Body Mass Index (BMI) of less than 30 kg/m^2^. A BMI greater than 30 kg/m^2^ is a surgical contraindication to the DAA approach for THA surgery and affects the visualization during the DAA approach; (6) Absence of significant deformities or defects in the pelvic bone structure. The exclusion criteria encompassed: (1) Inability to withstand surgical and anesthetic risks, presence of mental health disorders, or incapacity to engage in postoperative rehabilitation; (2) BMI of 30 kg/m^2^ or greater; (3) Bone metabolic disorders, rheumatoid arthritis, severe osteoporosis, or bone tumors; (4) Incomplete clinical data, with follow-up duration of less than 12 months; (5) Absence of preoperative planning utilizing the AI-HIP system. Following the screening process, 287 patients were identified and included in this study, with all surgical procedures conducted by a single, highly experienced lead surgeon. Comprehensive baseline information is provided in Table 1.

**TABLE 1:** Demographic and preoperative data.

Item	DAA	PLA	*P*
Gender^a^			0.168
Male	98	38	
Femle	108	43	
Age^b^	62.16 ± 14.19	64.11 ± 12.75	0.282
BMI^b^	24.21 ± 3.35	24.56 ± 3.15	0.424
Operation side^a^			0.5
Left	101	42	
Right	105	39	
surgical indication			0.45
OA	48	17	
ONFH	62	24	
FNF	40	14	
DDH	54	26	

OA, osteoarthritis; ONFH, osteonecrosis of the femoral head; FNF, Famoral neck fracture; DDH, Developmental dysplasia of the hip.

^a^ Data are presented as n.

^b^ Data are presented as mean.

#### 2.1.2 Choice of surgical method

In our study on total hip arthroplasty (THA), the selection of the surgical approach was guided by specific criteria to ensure optimal patient outcomes. All participants met the eligibility requirements for both the Direct Anterior Approach (DAA) and the posterolateral approach (PLA), allowing for an unbiased comparison between the two methods.

(1) Patient Eligibility: Each patient was thoroughly evaluated to confirm their suitability for both DAA and PLA, ensuring that they met all clinical indications necessary for either approach.
(2) Informed Decision-Making: Comprehensive preoperative education was provided to all patients, detailing the benefits, risks, and expected recovery profiles of each surgical method. This standardized education ensured that patients received consistent and accurate information.
(3) Autonomous Choice: Patients who demonstrated the capacity for informed decision-making were empowered to select their preferred surgical approach. This choice was made following detailed counseling and was formalized through the signing of an informed consent form.
(4) Study Enrollment: Of the 287 patients enrolled, 206 selected the DAA, while 81 opted for the PLA. This distribution highlights patient preferences when informed consent and autonomy are prioritized in the decision-making process.

### 2.2 Preoperative planning

Preoperative planning in this study was performed by the surgeon preoperatively by uploading the hip and knee joint scan to the AI-HIP preoperative planning system. The AI-HIP system is founded on the principles of deep learning and intelligent planning, facilitating the swift identification, rectification, and assessment of data through artificial intelligence, thus obviating the necessity for manual segmentation of computed tomography (CT) images. This system is capable of autonomously correcting pelvic images and is designed to be user-friendly (Maria et al., 2020). The procedural steps involved are as follows.

Preoperative scanning was performed using a low-dose CT program (specific parameters: electron tube voltage 100 kVp, electron tube current 20 mA, slice thickness 1 mm, voxel size 0.34 mm × 0.34 mm × 0.6 mm). The scanning area included only the hip and knee joints on the operated side, thus avoiding whole-body radiation in order to minimize the radiation exposure to the patient.

**FIGURE 1:**
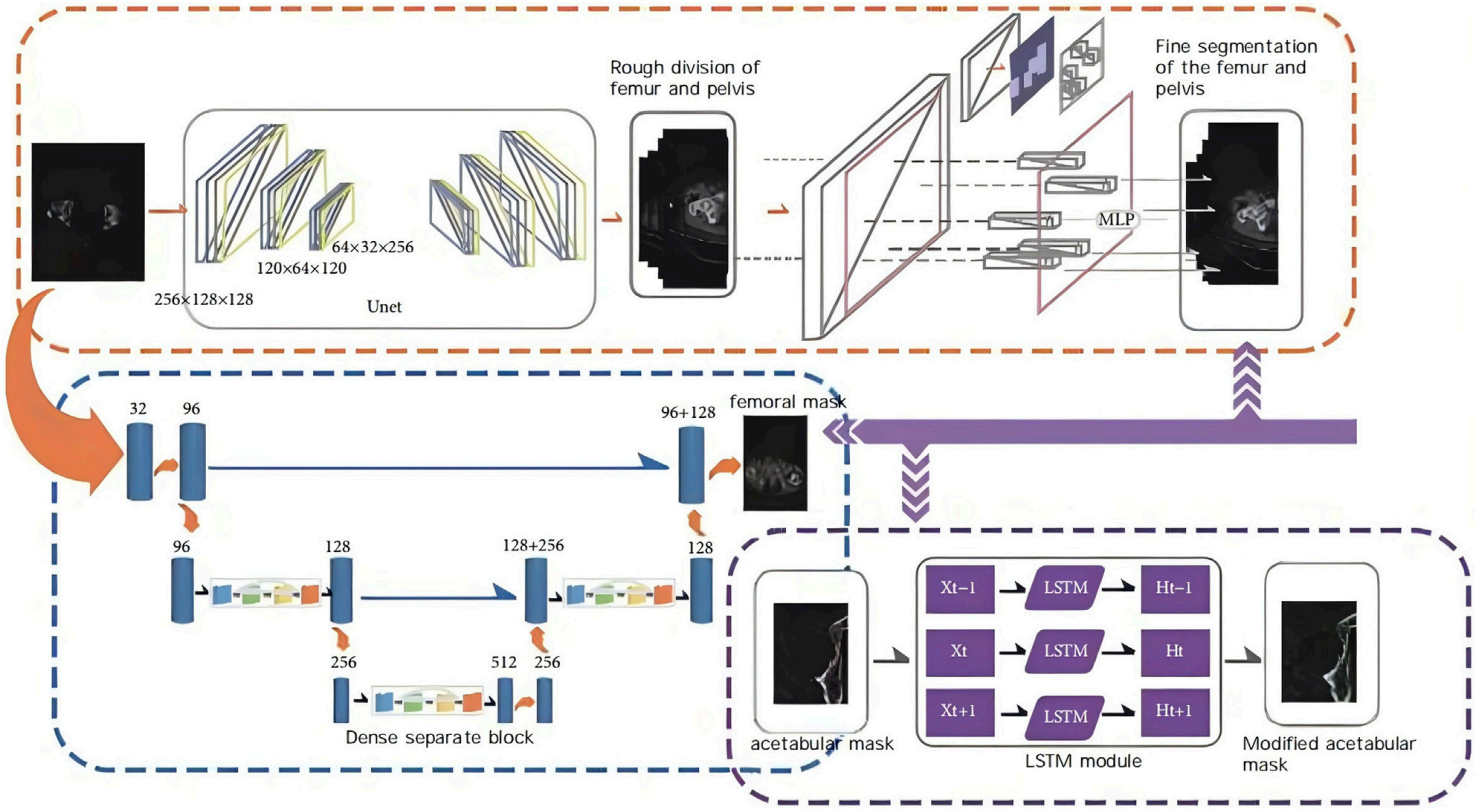
Schematic diagram of AIHIP self-developed unique algorithm (G-NET net-work) for skeletal segmentation.

Import the scanned data in DICOM format into the AI-HIP software, and perform three-dimensional reconstruction of the hip joint utilizing the Transformer-unet algorithm (illustrated in Figure 1).

Utilize the AI-HIP system to execute intelligent planning for both the femoral and acetabular components. For the acetabular side, position the acetabular cup prosthesis with an abduction angle of 40° and a cup anteversion of 20°. Based on the preoperative planning for the patient’s FNA, predict the suitable FNA implant for the femoral stem, and subsequently select an appropriate ball head based on the reconstructed model to finalize the simulated prosthesis placement.

Implement intelligent bone sawing simulation to ascertain the length of the femoral segment to be preserved and the distance from the tip of the greater trochanter to the shoulder of the femoral stem, ultimately producing the intelligent planning results that simulate the postoperative outcome.

**FIGURE 2:**
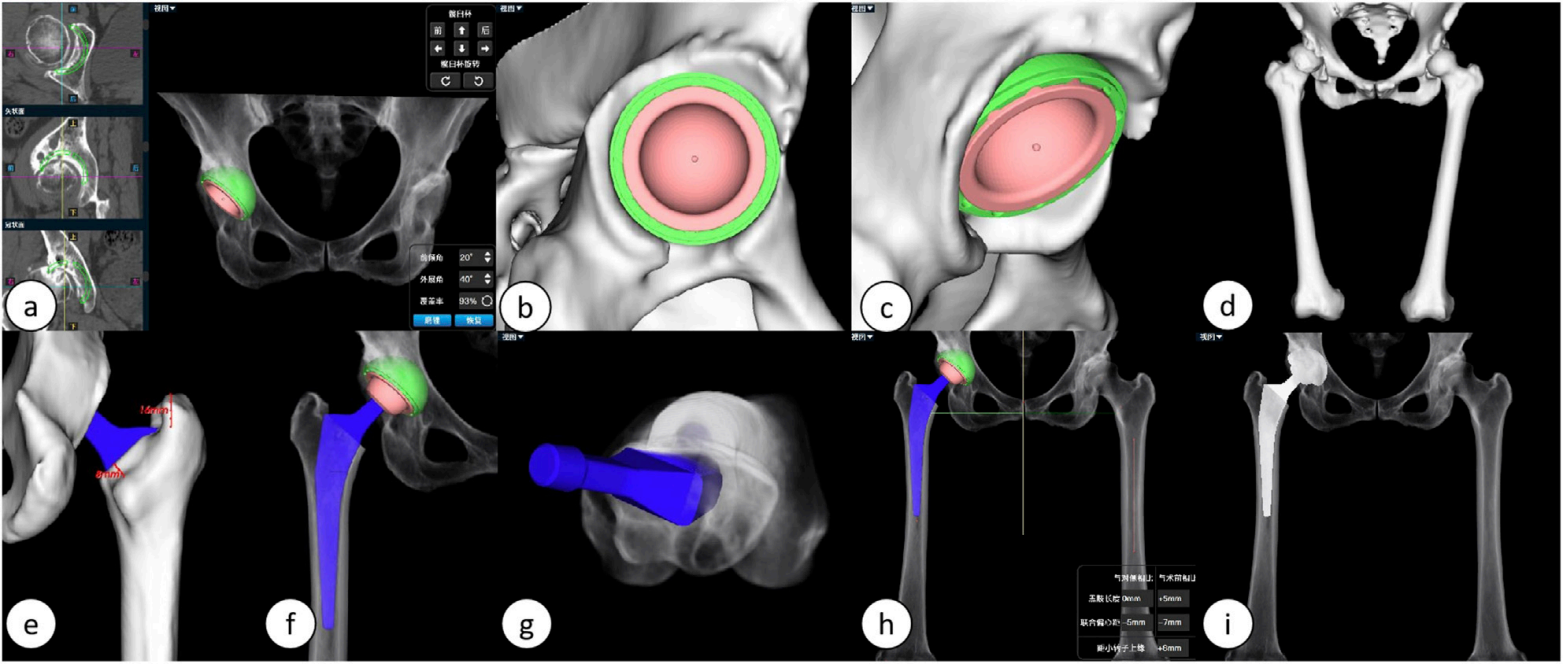
Illustrates the preoperative planning process using the AIHIP system: **(A)** The AI calculates the optimal placement position for the acetabular cup. **(B, C)** Detailed views of the acetabular side. **(D)** Three-dimensional reconstruction of the lower limb. **(E, F)** Detailed views of the femoral stem. **(G)** Predicted postoperative femoral stem anteversion angle. **(H)** Simulated postoperative overall three-dimensional view. **(I)** Simulated postoperative anteroposterior X-ray of the hip joint.

The duration required for preoperative planning using the AI-HIP system is approximately 5 minutes, with the results of the preoperative planning illustrated in Figure 2.

### 2.3 Surgical technique

The surgical procedure was performed by a single, highly skilled surgeon affiliated with the Department of Orthopedics at the Jiangsu Provincial Hospital of Traditional Chinese Medicine. The operation employed a total hip joint system, comprising the acetabular Pinnacle cup and the femoral Corail stem, both produced by the American corporation Johnson and Johnson (DePuy). After the administration of general anaesthesia, both the DAA approach and the PLA approach were performed in the lateral position. This position was chosen to optimise surgical access and visualisation for both approaches and to ensure consistency across procedures.

**FIGURE 3:**
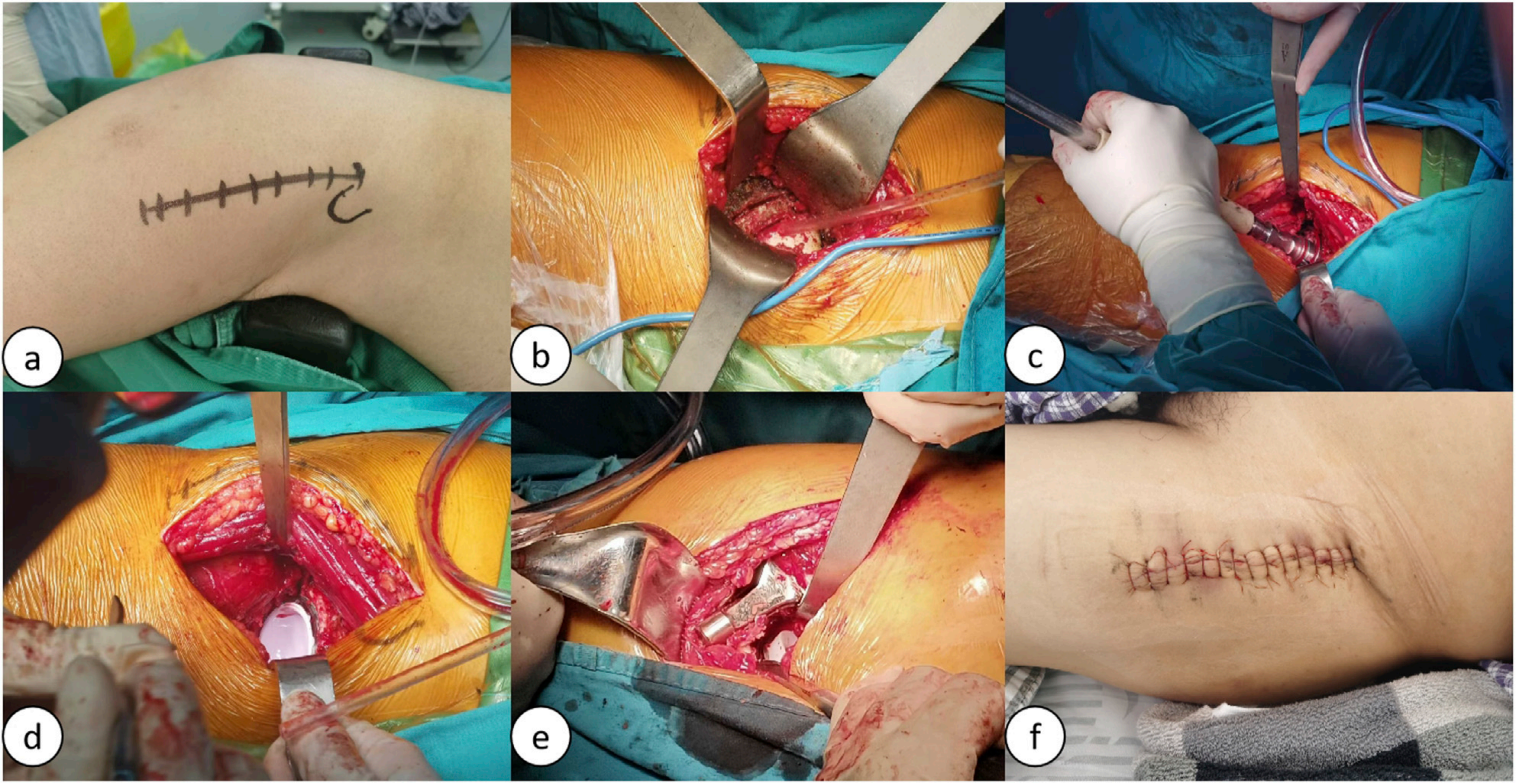
Schematic of the lateral decubitus position DAA procedure: **(A)** Preoperative markings for the DAA procedure; **(B)** Exposure of the joint cavity, osteotomy, and removal of the femoral head; **(C)** Reaming of the acetabulum; **(D)** Insertion of the acetabular prosthesis; **(E)** Canal broaching and insertion of the femoral stem prosthesis; **(F)** Suture incision.

(1) DAA: The acetabulum should be exposed through the muscle gap (the gap between the broad fascia tensor/suture muscles and the gluteus medius), and direct observation of the bony landmarks on the anterior wall of the acetabulum (e.g., anterior inferior iliac spine, anterior edge of the transverse acetabular ligament) should be emphasized; however, due to the anterior capsule tension, the anterior angle of the acetabular filing should be finely controlled (15°–20° is recommended) in order to avoid insufficient anterior tilt. Because of the limited proximal femoral distraction, medullary preparation is prone to excessive anterior tilt of the prosthesis (requiring external rotation of the lower extremity and posterior extension of the hip), and internal rotation of the femoral stem needs to be avoided (Dargel et al., 2014; Lawrence and John, 2019). The DAA approach surgical procedure is shown in Figure 3.
(2) PLA: The posterior lateral approach enters through the gap between the gluteus maximus and the external rotator group, and needs to cut off part of the external rotator group, with the transverse acetabular ligament and sciatic tuberosity as the reference for posterior tilt; however, posterior displacement of the femur may obscure the acetabular view, and needs to be combined with the femoral lateral trial mold for temporary reset to assist in localization. The lateral femur is adequately exposed, but insufficient soft tissue release on the posterior side may lead to posterior tilt of the femoral stem, and balance stability should be partially preserved by the posterior joint capsule.

### 2.4 Perioperative management

During the 24-h postoperative period, the patient’s vital signs are meticulously monitored, with a specific focus on the recovery of consciousness, sensory perception, and motor function on the surgical side. Simultaneously, patients receive a regimen that includes pantoprazole for gastric protection, ambroxol to aid in expectoration, citrus flavonoids to mitigate edema, flurbiprofen axetil for analgesia, ceftriaxone sodium for antibacterial prophylaxis, and enoxaparin sodium for anticoagulation purposes. For patients diagnosed with osteoporosis, denosumab injections are administered as clinically indicated. From the first to the third postoperative day, patients are instructed to engage in ankle pump exercises, leg raises, ambulation with crutches, and active flexion and extension exercises to facilitate recovery. The overarching objective is to enable patients to walk independently without the assistance of crutches and to achieve the capacity to bear weight effectively (Luc et al., 2020).

### 2.5 Efficacy indices

**FIGURE 4:**
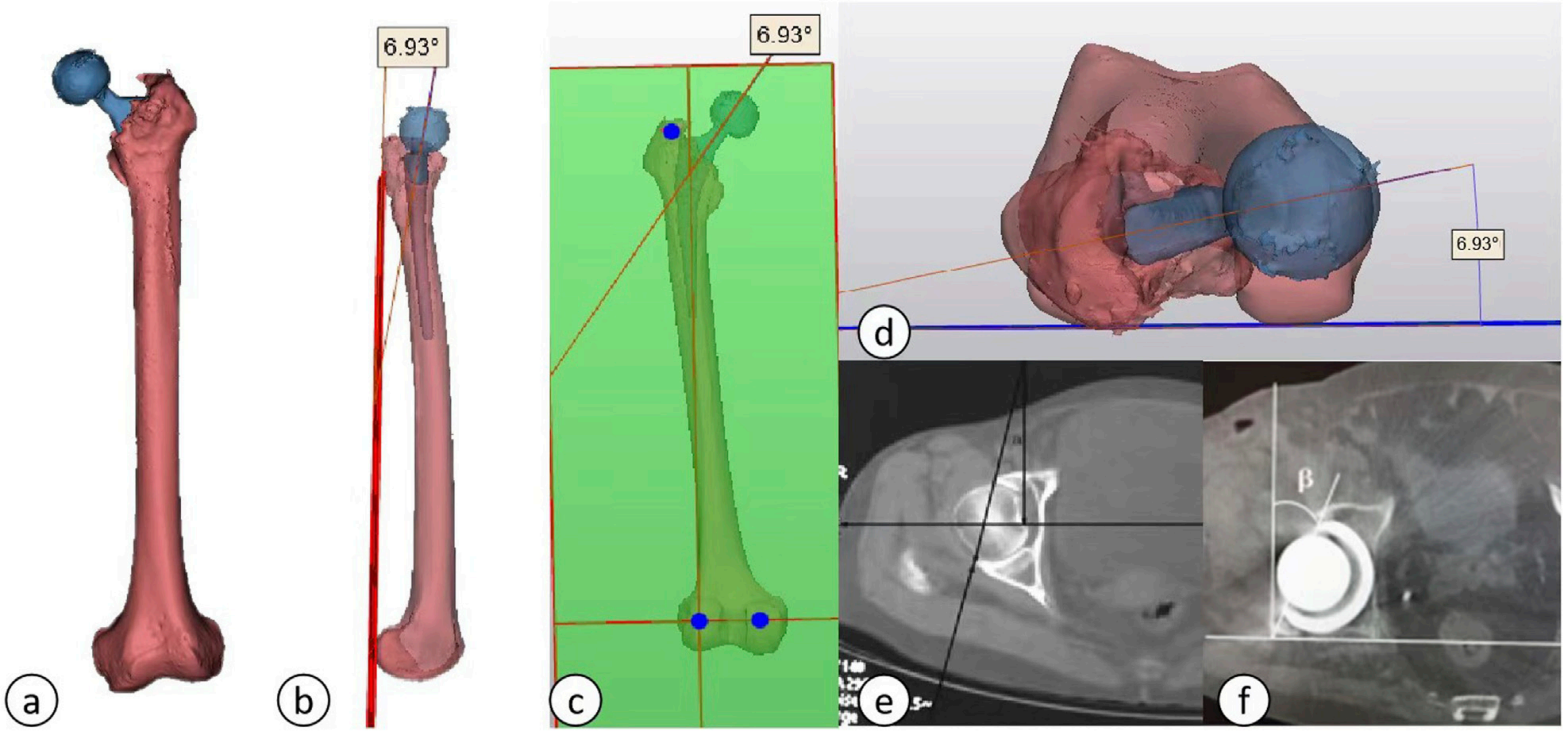
**(A)** Three-dimensional reconstruction model of the femoral shaft and femoral neck; **(B–D)**: Different angled views of acetabular anteversion; **(E)** Preoperative anterior acetabular tilt angle; **(F)** Postoperative cup anteversion angle of the acetabular prosthesis.

**FIGURE 5:**
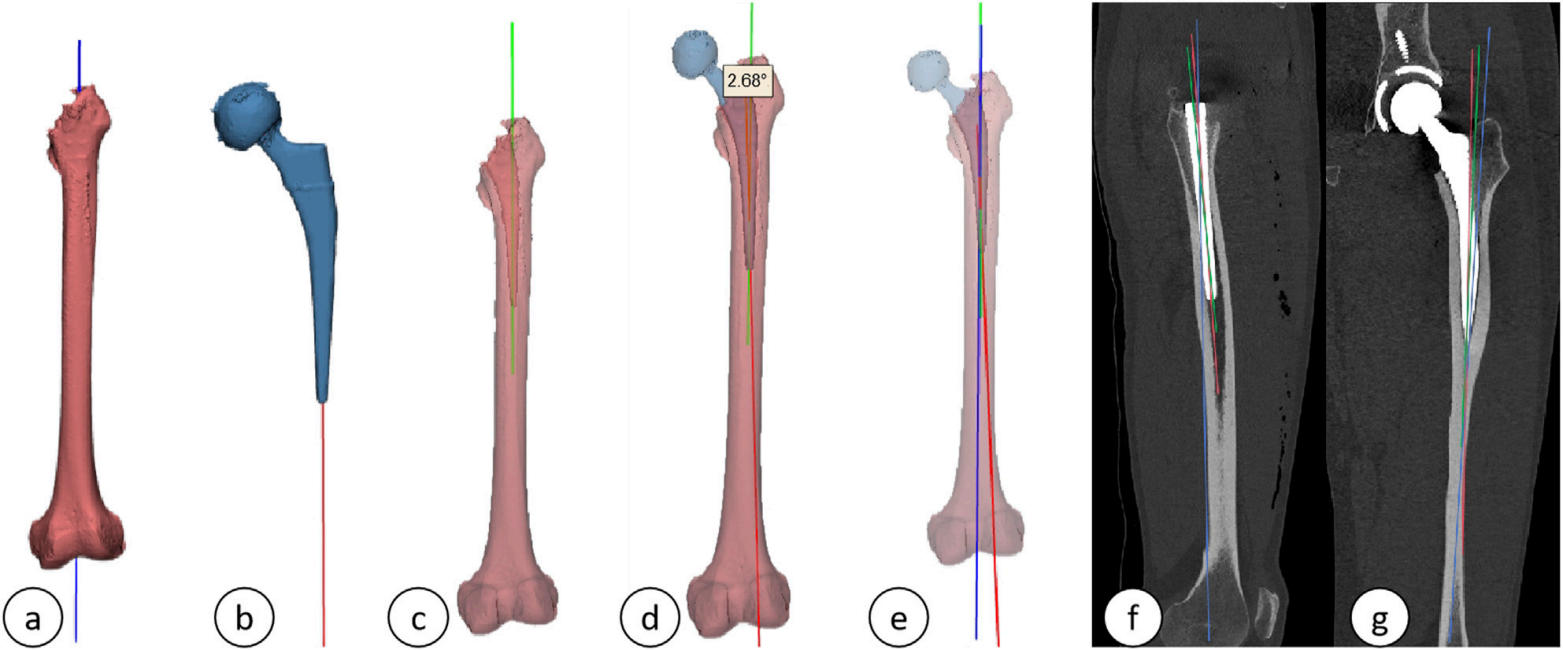
**(A)** Axis of the femur; **(B)** Axis of the femoral stem prosthesis; **(C)** Proximal femoral axis; **(D)** Alignment; **(E)** Three-dimensional schematic of the axes (green: proximal femoral axis; red: axis of the femoral stem prosthesis; blue: femoral axis); **(F, G)** Sagittal and coronal plane projections.

(1) Radiological Assessment Indices: All patients underwent de-noised computed tomography (CT) scans utilizing the Siemens SENSATION 256-slice spiral CT prior to discharge on the third postoperative day. The scanning protocol encompassed a range from the pelvis to the proximal femur, with a slice thickness of 0.64 mm. The acquired scan data were stored in DICOM format and subsequently recorded onto discs. Both preoperative and postoperative CT scans were imported into Mimics software (version 21.0, Materialise, Leuven, Belgium) in DICOM format, facilitating the three-dimensional reconstruction of the femoral stem and shaft. The reconstructed data were then transferred to the 3-matic Research software (version 18.0, Materialise, Leuven, Belgium) for further analysis.
(2) The following parameters were measured and documented: ① Femoral (prosthetic) neck anteversion (FNA): This parameter was defined as the angle between the surgical transepicondylar line—established by connecting the most prominent point of the lateral condyle and the deep groove of the medial condyle—and the axis of the femoral neck/stem neck. The axis of the femoral neck (prosthesis) was determined using the Design function of the 3-matic Research software. A plane was established based on the lateral and medial aspects of the distal femoral condyles and the lateral tuberosity, referred to as the posterior condylar plane. The angle between the axis of the femoral neck (prosthesis) and the posterior condylar plane was measured using the Measure tool in the 3-matic Research software, thereby providing a precise calculation of the anteversion angle, as depicted in Figures 4A–D. ② Acetabular (prosthetic) anteversion (AA): This was quantified as the angle between the lines connecting the superior and inferior margins of the acetabulum and the bilaterally lateral tears, Figure 4E shows preoperative acetabular anterior inclination and Figure 4F shows postoperative acetabular prosthesis anterior inclination Figure 4F. ③ Alignment: This parameter was defined as the angle between the distal axis of the femoral stem and the proximal axis of the femur. The axis of the femoral stem was selected for the prosthetic distal component, and the fitting procedure was executed using the Analytical command within the Design function of the 3-matic Research software. The proximal axis of the femur was characterized as a line connecting the midpoint of the horizontal canal of the lesser trochanter to the midpoint of the canal of the femoral trochanter, as shown in Figures 5A–E. ④ Femoral stem coronal alignment measurement: The femoral anatomical axis was assessed due to the anterior curvature of the femoral shaft and notable anatomical variations. The fitting procedure employed the Analytical command within the Design function of the 3-matic Research software to fit inertia axes. On the coronal plane, the angle between the axis of the femoral stem and the femoral axis provided the coronal alignment angle of the femoral stem, while the angle between the proximal axis of the femur and the femoral anatomical axis yielded the coronal inclination angle of the femur (Hirohito et al., 2015; Zurmuehle et al., 2017). A positive value indicated valgus alignment, whereas a negative value indicated varus insertion. An angle between −3° and 3° was classified as neutral insertion (Hirohito et al., 2015). The specific alignment is illustrated in Figure 5G. ⑤ The measurement of femoral stem sagittal alignment was conducted in a manner analogous to that of coronal alignment assessment. Specifically, the sagittal alignment of the femoral stem is characterized by the difference between the sagittal inclination angle of the femoral stem and that of the femur. A negative value of less than −3° is classified as a flexed insertion of the femoral prosthesis, while a positive value exceeding 3° is categorized as an extended insertion. Values falling between −3° and 3° are considered to represent neutral insertion (Hirohito et al., 2015; Zurmuehle et al., 2017). The details of the specific sagittal alignment are depicted in Figure 5F.

To ensure the reliability of the data, all measurements were conducted by two observers using the same methodology. These measurements were repeated at 1-month intervals, and inter-rater reliability was assessed using the intraclass correlation coefficient (ICC), with mean values calculated for analysis.

**FIGURE 6:**
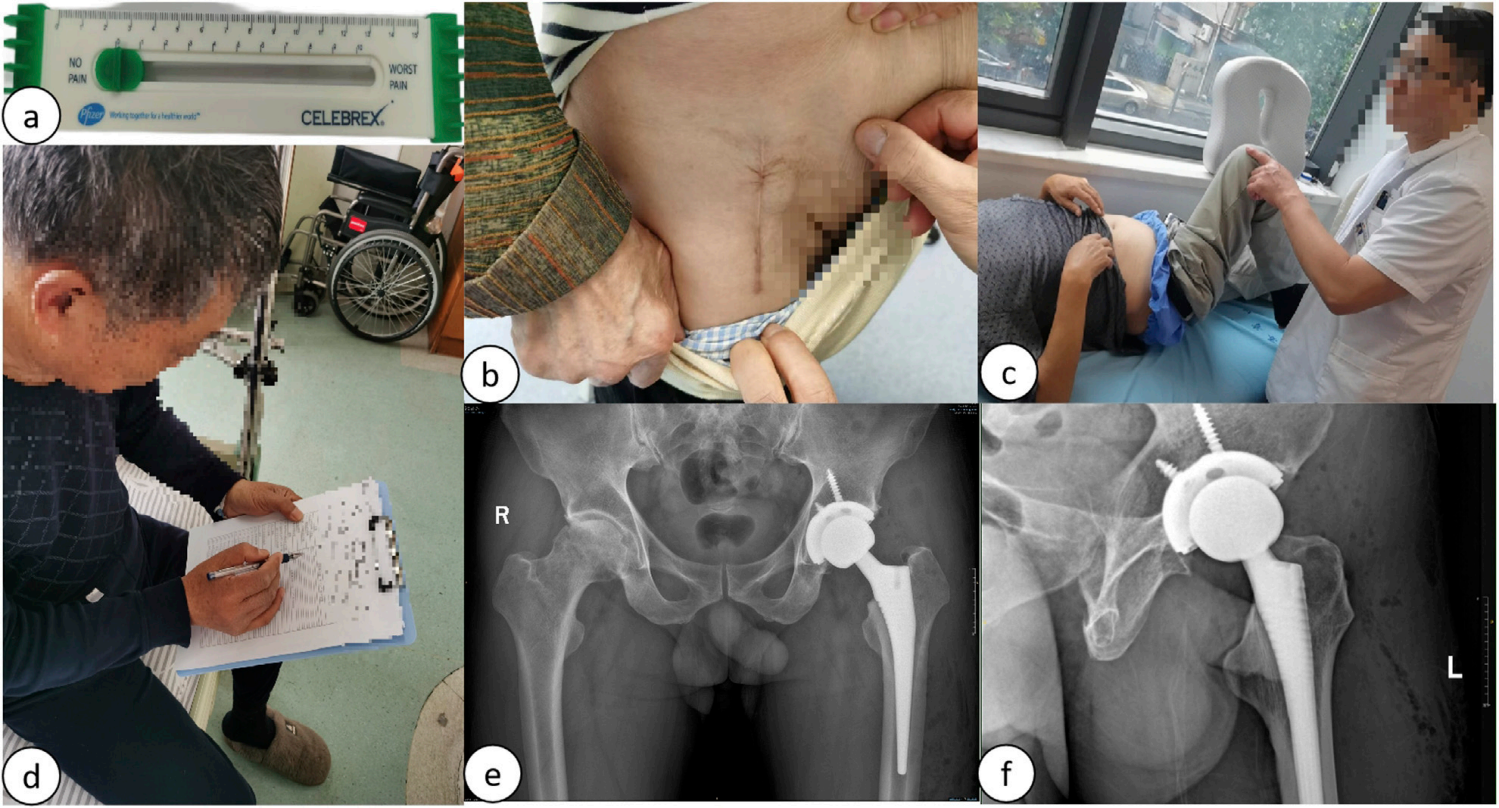
**(A)** VAS score measurement; **(B)** Follow-up of incision scars 6 months postoperatively; **(C)** Follow-up of functional range of motion 6 months postoperatively; **(D)** Follow-up of postoperative Harris Hip and WOMAC scores; **(E)** Follow-up of postoperative bilateral hip joint anteroposterior radiographs; **(F)** Follow-up of postoperative hip joint lateral radiographs.

**FIGURE 7:**
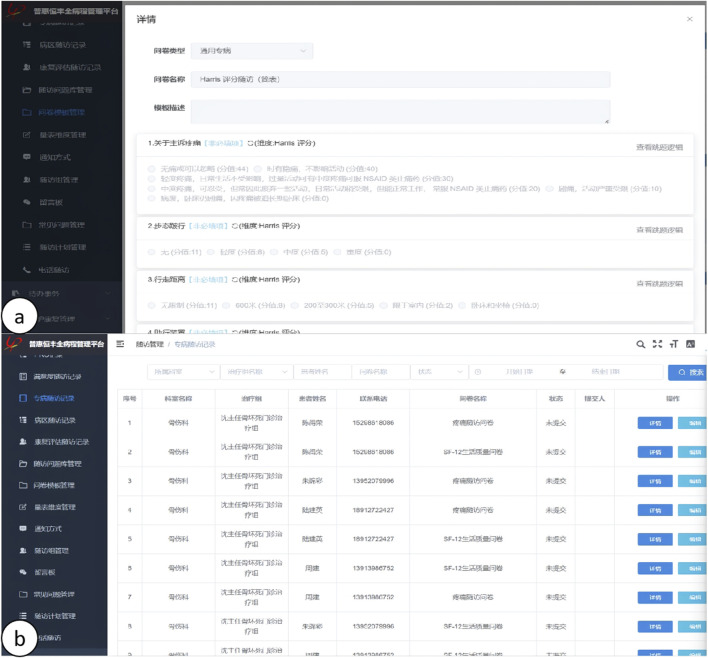
**(A, B)** Illustration of the online long-term follow-up on the HP CareCycle Management Platform.

(1) Efficacy Indices: Postoperative functional recovery was evaluated utilizing the Hip Joint Harris Score and the Western Ontario and McMaster Universities Osteoarthritis Index (WOMAC Score). Elevated Harris Scores and diminished WOMAC Scores were indicative of enhanced postoperative recovery. The hip joint Harris Scores and WOMAC Scores for both cohorts were recorded prior to surgery and subsequently at 1 week, 4 weeks, 3 months, and 6 months postoperatively. The Visual Analog Scale (VAS) was utilized to assess pain levels, with VAS Scores documented at 24 h, 72 h, 1 week, 4 weeks, 3 months, and 6 months following the surgical procedure. Hemoglobin (Hb) levels were measured as an indicator of blood loss during the operation, with Hb levels recorded and subjected to statistical analysis at preoperative, first postoperative day, third postoperative day, and first postoperative month intervals. Additionally, levels of creatine kinase (CK), C-reactive protein (CRP), and interleukin-6 (IL-6) were quantified to evaluate the degree of tissue injury, with CK, CRP, and IL-6 levels recorded and statistically analyzed at preoperative and postoperative intervals of 2 h, 1 day, 3 days, and 1 week. Furthermore, the length of the surgical incision, duration of the surgery, and postoperative hospital stay were documented and statistically analyzed. Follow-up assessments were conducted post-discharge through a comprehensive disease management platform, as depicted in Figures 6, 7.
(2) Complications: The occurrence of postoperative adverse events, including sensory abnormalities, intraoperative fractures, delayed wound healing, postoperative infections, dislocations, and the formation of deep vein thrombosis (DVT), was carefully monitored. All clinical and follow-up data were recorded by two independent researchers who did not participate in the surgical procedures.

## 3 Statistical methods

Statistical analyses were conducted using IBM SPSS Statistics software, version 27.0.1. Descriptive analysis was performed on continuous data, which are presented as mean ± standard deviation (x ± s). Independent sample t-tests were employed to compare differences between the two groups for Fine Needle Aspirations (FNA), Arthrocentesis (AA), changes from preoperative to postoperative FNA and AA values, the Harris score, the VAS score, hemoglobin (Hb) levels, incision length, surgical duration, hospital stay, time to ambulation postoperatively, and the level of significance (P-value = 0.05). Chi-square tests were used to compare the distribution of patient sex, surgical side, indications for surgery, incidence of DVT, rate of dislocation, and infection rates between the two groups.

## 4 Results

In this investigation, a total of 271 patients, encompassing 287 hips, were monitored, with follow-up durations extending a minimum of 6 months postoperatively. The differences in preoperative femoral anterior angulation (FNA) and acetabular anterior angulation (AA) were not significantly different (p > 0.05), the length of incision in patients in the DAA group was significantly shorter than that in the PLA group (DAA:10.64 ± 0.941 vs. PLA:15.21 ± 1.325, p < 0.001), and the duration of operation in patients in the DAA group was significantly longer than that in the PLA group (DAA. 118.67 ± 26.95 VS PLA:53.27 ± 58.71, p < 0.001), hospitalization time of patients in DAA group was significantly shorter than that of PLA group (DAA:7.59 ± 4.18 VS PLA:9.09 ± 3.65, p < 0.001), and early postoperative time to get down to the floor of the floor of the patients in DAA group was significantly earlier than that of the patients in PLA group (1.03 ± 0.16 vs. 1.18 ± 0.41, p = 0.05); postoperative FNA (DAA: 12.97 ± 6.93 vs. PLA:10.31 ± 9.46, p = 0.009) and postoperative AA (DAA: 20.93 ± 7.54 vs. PLA: 24.34 ± 7.93, p < 0.001), which were significantly different; and postoperative acetabular abduction angle (DAA: 44.52 ± 3.52 vs. PLA: 45.19 ± 4.02, p = 0.185) and postoperative CA (DAA: 32.75 ± 9.92 vs. PLA: 32.90 ± 12.72, p = 0.915), which were not significantly different; and the difference between preoperative AI-predicted FNA and postoperative FNA (DAA: 3.12 ± 5.88 vs. PLA: 5.59 ± 8.21, p = 0.005) and the difference between preoperative AI-predicted AA and postoperative AA (DAA: 0.93 ± 7.54 vs. PLA: 4.34 ± 7.93, p < 0.001) were significantly different, and the angle between the distal shaft of the femoral stem and the proximal shaft of the femur (DAA: 2.87 ± 1.86 vs. PLA: 2.62 ± 1.94, p = 0.001) was significantly different. 1.94, p = 0.435) and the angle between the coronal projection of the distal axis of the femoral stem and the proximal axis of the femur (DAA: 1.85 ± 0.76 vs. PLA: 1.74 ± 0.85, p = 0.522) and the angle between the sagittal projection of the distal axis of the femoral stem and the proximal axis of the femur (DAA: 2.20 ± 0.51 vs. PLA: 1.95 ± 0.64, p = 0.561) were not significantly different. Detailed findings are presented in Table 2.

**TABLE 2:** Comparison of clinical data between the DAA group and the PLA group.

Item	DAA (n = 206)	PLA(n = 81)	t	P
Incision length (cm)	10.64 ± 0.941	15.21 ± 1.325	−2.12	<0.001
Surgical time (min)	118.67 ± 26.95	53.27 ± 58.71	−3.55	<0.001
Length of hospital stay (d)	7.59 ± 4.18	9.09 ± 3.65	−0.38	<0.001
Time to weight bearing postoperatively (d)	1.03 ± 0.16	1.18 ± 0.41	−2.51	0.050
Preoperative FNA(°)	17.75 ± 8.71	18.21 ± 10.13	−0.23	0.821
Preoperative AA (°)	20.68 ± 6.48	21.57 ± 8.68	−0.944	0.346
Postoperative FNA (°)	12.97 ± 6.93	10.31 ± 9.46	2.62	0.009
Difference between preoperative AI predicted FNA and postoperative FNA (°)	3.12 ± 5.88	5.59 ± 8.21	−2.845	0.005
Postoperative AA (°)	20.93 ± 7.54	24.34 ± 7.93	−3.401	<0.001
Difference between preoperative AI predicted AA and postoperative AA (°)	−0.93 ± 7.54	−4.34 ± 7.93	3.401	<0.001
CA (°)	32.75 ± 9.92	32.90 ± 12.72	−0.107	0.915
Acetabular abduction (°)	44.52 ± 3.52	45.19 ± 4.02	1.459	0.185
Angle between the axis of the distal femoral stalk and the axis of the proximal femur (°)	2.87 ± 1.86	2.62 ± 1.94	0.782	0.435
Angle between the projection of the distal axis of the femoral stalk and the proximal axis of the femur in the coronal plane (°)	1.85 ± 0.76	1.74 ± 0.85	0.568	0.522
Angle between the projection of the distal femoral stem axis and the proximal femoral axis in the sagittal plane (°)	2.20 ± 0.51	1.95 ± 0.64	0.621	0.561

**FIGURE 8:**
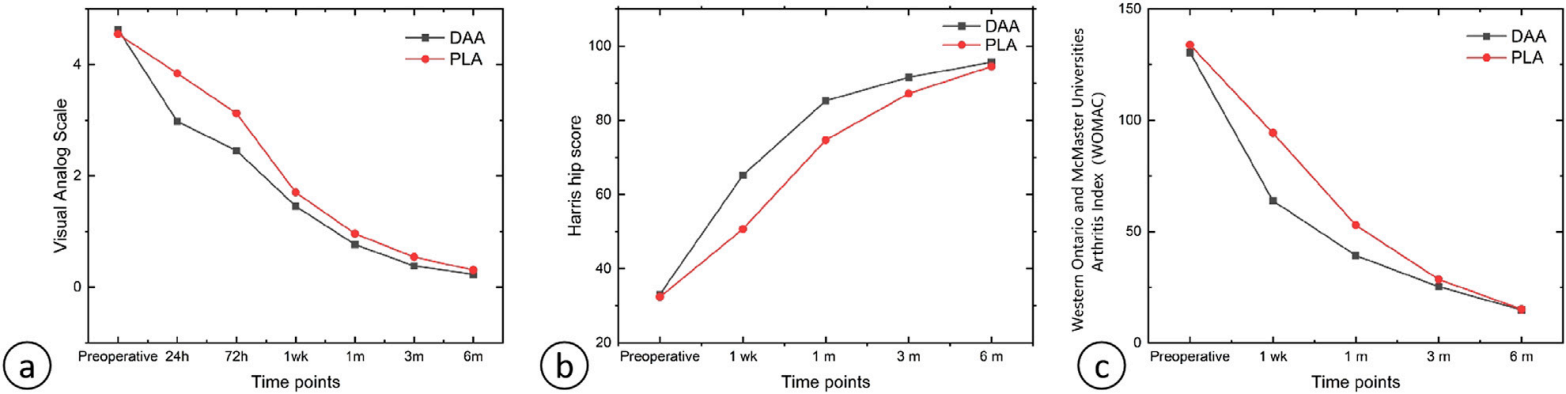
**(A)** Line graph of VAS scores; **(B)** Line graph of Harris Hip Scores; **(C)** Line graph of WOMAC scores.

There were no significant differences in the levels of hemoglobin (Hb), creatine kinase (CK), C-reactive protein (CRP), and interleukin-6 (IL-6) between the two groups at 2 h, 1 day, 3 days, 1 week, and 4 weeks post-surgery (P > 0.05), as depicted in Tables 3, 4. Postoperative Visual Analog Scale (VAS) scores for both groups were significantly lower than their preoperative counterparts, with the VAS scores of the DAA group being lower than those of the PLA group at 24 h, 72 h, 1 week, 4 weeks, and 3 months postoperatively, demonstrating a statistically significant difference (P < 0.05), as illustrated in Table 5 and Figure 8. The Harris scores for the DAA group were significantly higher than those for the PLA group at both 4 weeks and 3 months postoperatively (P < 0.05), while the Western Ontario and McMaster Universities Osteoarthritis Index (WOMAC) scores in the DAA group were significantly lower than those in the PLA group at 1 and 4 weeks postoperatively (P < 0.05), as indicated in Table 6. No statistically significant differences were observed in the comparison of VAS scores, WOMAC scores, and Harris scores between the two groups at the 6-month postoperative mark (P > 0.05).

**TABLE 3:** Comparison of postoperative serological indicators between two approaches.

Approach	CK(iu/L)			CRP (mg/dL)		
	DAA	PLA	P	DAA	PLA	P
Time points						
2 h postoperative	312.03 ± 63.61	315.81 ± 56.62	0.640	11.87 ± 6.15	12.69 ± 7.36	0.335
1 day postoperative	493.70 ± 54.26	503.62 ± 61.17	0.180	25.49 ± 9.87	27.24 ± 14.01	0.233
3 days postoperative	170.13 ± 43.51	179.95 ± 42.20	0.084	33.36 ± 13.41	36.01 ± 18.71	0.182
1 week postoperative	136.19 ± 61.74	138.35 ± 52.61	0.782	12.38 ± 4.53	12.84 ± 6.10	0.484
4 weeks postoperative	127.26 ± 61.43	129.04 ± 69.96	0.832	9.49 ± 4.63	10.31 ± 5.04	0.199

**TABLE 4:** Comparison of hemoglobin and Interleukin-6 levels at different time points between two approaches.

Approach	Hb(g/L)			IL-6 (pg/mL)		
	DAA	PLA	P	DAA	PLA	P
Time points						
Preoperative	132.15 ± 15.29	131.20 ± 16.869	0.649	-	-	-
2 h postoperative	-	-	-	37.75 ± 17.89	34.63 ± 15.57	0.171
1 day postoperative	116.61 ± 14.81	114.31 ± 12.415	0.221	144.01 ± 39.66	138.89 ± 36.56	0.420
3 days postoperative	106.20 ± 13.865	103.19 ± 10.329	0.079	37.82 ± 21.58	40.20 ± 19.52	0.388
1 week postoperative	114.84 ± 17.118	119.81 ± 16.531	0.061	10.80 ± 4.79	11.42 ± 4.95	0.330
4 weeks postoperative	127.18 ± 14.349	125.65 ± 13.612	0.413	5.45 ± 3.18	5.13 ± 2.94	0.442

**TABLE 5:** Comparison of VAS scores at each time point between the DAA group and the PLA group.

Time points	DAA	PLA	P
Preoperative	4.63 ± 0.83	4.55 ± 0.69	0.550
24 h postoperative	2.978 ± 0.768	3.943 ± 1.001	<0.001
72 h postoperative	2.456 ± 0.743	3.126 ± 0.771	<0.001
1week postoperative	1.456 ± 0.818	1.704 ± 0.843	0.023 (<0.05)
1 m postoperative	0.767 ± 0.694	0.963 ± 0.782	0.039 (<0.05)
3 m postoperative	0.384 ± 0.553	0.543 ± 0.653	0.038 (<0.05)
6 m postoperative	0.233 ± 0.457	0.309 ± 0.516	0.225 (>0.05)

**TABLE 6:** Comparison of harris scores and WOMAC scores at different time points between two approaches.

Approach	Harris scores			WOMAC scores		
	DAA	PLA	P	DAA	PLA	P
Time points						
Preoperative	33.14 ± 8.68	32.41 ± 10.93	0.348	130.43 ± 33.52	133.85 ± 27.43	0.564
1 week postoperative	65.21 ± 5.67	50.68 ± 8.62	<0.001	63.88 ± 21.646	94.33 ± 19.662	<0.001
4 weeks postoperative	85.26 ± 7.36	74.67 ± 9.74	<0.001	39.311 ± 13.56	52.938 ± 17.992	<0.001
3 m postoperative	91.58 ± 3.86	87.20 ± 1.75	0.012	25.276 ± 15.812	28.550 ± 14.132	0.107
6 m postoperative	95.65 ± 2.84	94.52 ± 2.23	0.642	14.854 ± 7.757	15.15 ± 8.868	0.781

Within the DAA group, two patients experienced postoperative hip dislocation, and seven patients developed postoperative infections (three deep infections and four superficial infections). These cases were managed through one-stage and two-stage revision surgeries, guided by bacterial culture and macro gene detection, ultimately resulting in the resolution of the infections. The remaining patients in this cohort did not encounter any complications. In the PLA group, two patients experienced postoperative hip dislocation, and two patients developed postoperative infections (one deep infection and one superficial infection), both of which were successfully addressed through revision surgery. The analysis revealed no statistically significant difference in early complications between the two groups (P > 0.05), as presented in Table 7.

**TABLE 7:** Comparison of postoperative complication rates between the DAA group and the PLA group.

Groups	Example number	The prosthesis loose	Lower limbs DVT	Dislocation of the hip joint	Deep infection	Surface infection	Incidence rate
DAA	206	0	0	2 (0.97%)	3 (1.46%)	4 (1.94%)	4.37%
PLA	81	0	0	2 (2.47%)	1 (1.23%)	1 (1.23%)	4.94%
*P*	-	-	-	0.570	0.885	0.698	-

## 5 Discussion

This prospective study involving 287 patients indicates that individuals who underwent the DAA demonstrated significantly lower VAS and Western Ontario and WOMAC scores in the early postoperative period when compared to those who received the PLA. Moreover, the DAA cohort achieved notably higher Harris hip scores. At the 6-month follow-up, outcomes for both groups began to converge. Additionally, patients in the DAA group experienced shorter hospital stays and earlier ambulation following surgery, suggesting that the DAA is associated with reduced postoperative pain, decreased duration of hospitalization, and expedited recovery (Higgins et al., 2015; Jetse et al., 2017). Mao et al. found (Jin et al. 2023) that while the DAA resulted in longer operative times, it also led to reduced intraoperative bleeding and muscle damage. At 3 months post-surgery, patients who underwent DAA reported lower VAS scores and higher Harris scores compared to those who underwent PLA, with the differences being statistically significant in favor of the DAA group. Another research team (Sarhan et al., 2024) conducted a retrospective analysis of 731 DAA cases compared to 840 PLA cases in THA and found results consistent with those of the current study. The DAA approach was associated with shorter hospital stays, reduced pain following total hip replacement, accelerated recovery, and lower healthcare costs (Tze et al., 2017). Furthermore, the incision length for the DAA was significantly shorter than that for the PLA.

A study (McMullen et al., 2018) indicated that the smaller incision and soft tissue release associated with the DAA, along with the challenges in preparing the lateral femur, are likely to contribute to suboptimal prosthesis placement. In contrast, the presence of an intraoperative assistant during the PLA can facilitate the accurate positioning of the femoral stem. The placement of the femoral stem in the DAA is predominantly reliant on the operator’s judgment, which may render it more vulnerable to alignment errors. Another investigation (Batailler et al., 2018) revealed that, unlike cemented femoral prostheses where anteversion is managed by the surgeon, the positioning of compression-fit femoral prostheses is dictated by the morphology of the proximal medullary cavity. This characteristic restores the femur’s “natural anteversion,” leading to an increase in anteversion that exhibits considerable variability and limited adjustability. CA, defined as the sum of FNA and AA, is critical for the functional recovery of the hip joint following THA (Jolles et al., 2002). Dorr et al. Found (Lawrence et al., 2009) that patients with posterior hip dislocations exhibited a CA exceeding 50°, suggesting that maintaining CA within a range of 25°–50° during surgery may mitigate the risk of postoperative hip dislocation. In the present study, FNA and AA were assessed through three-dimensional reconstruction of postoperative hip structures, which demonstrated reduced measurement error compared to two-dimensional computed tomography (Sangeux et al., 2015). The findings indicated no statistically significant difference in preoperative FNA and AA between the DAA and PLA groups; however, a significant difference was observed in postoperative FNA and AA. When comparing postoperative FNA and AA with the preoperative values for AI planning, the DAA group exhibited postoperative FNA and AA that were more closely aligned with the preoperative planning angles, with no significant difference in CA between the two groups. A prior study concluded (Hirohito et al., 2015) that preoperative FNA was the primary determinant of postoperative FNA; however, this study found no significant difference in preoperative FNA and AA between the two approaches, suggesting that the primary factor influencing postoperative FNA and AA was the surgical access method. The authors posited that the enhanced accuracy of FNA and AA in the DAA group may be attributed to reduced interference with surrounding soft tissues (such as muscles and tendons), which minimizes distortion and displacement of anatomical markers, thereby improving the precision of angular measurements. Additionally, the DAA aligns with the natural anatomical planes of the hip joint, facilitating more accurate measurements by the attending surgeon. Furthermore, the proficiency of the surgeon is likely to play a significant role in these outcomes.

The analysis of our current statistical data revealed no significant differences in alignment between the DAA and the PLA groups. Additionally, there was no notable difference in the rates of inversion and eversion during implantation, with the majority of prostheses being positioned in a neutral alignment. Previous research indicates that minor misalignments in the coronal plane do not adversely affect long-term patient outcomes (Nicolas et al., 2019). Conversely, it has been established that sagittal flexion positioning of the prosthesis is associated with an increased risk of postoperative pain and dislocation (Michael et al., 2010). However, another study suggests (Junya et al., 2018) that sagittal flexion placement may decrease the incidence of intraoperative fractures and limit the range of extension and external rotation of the joint prosthesis. In the present study, the sagittal alignment of the lateral DAA was predominantly neutral, and there were no significant differences observed between the two surgical approaches regarding the angles of flexion and extension placement.

The DAA is often regarded as a minimally invasive surgical technique that circumvents the need to incise muscle tissue (Ahmed et al., 2023). However, anatomical investigations have demonstrated (van Oldenrijk et al., 2010; Meneghini et al., 2006) that THA performed via the DAA can still inflict damage on the anterolateral muscles of the hip. This assertion is further supported by a prospective study (Knut et al., 2015) that identified injury to the tensor fascia lata during the surgical intervention. Despite the classification of these techniques as minimally invasive, muscle damage can occur as a result of stretching and inadvertent dissection during the procedure. Additionally, when compared to the PLA, which involves the transection of a portion of the muscle, the excessive stretching of muscles during the DAA may lead to a more pronounced elevation in postoperative CK levels (Knut et al., 2015; Kyrill et al., 2017). While the muscle separation and incision associated with the PLA may result in limited damage to individual muscle fibers, the stretching involved in the DAA may cause more extensive muscle injury (Knut et al., 2015). In the present study, no significant differences were observed between the two surgical groups regarding CK, IL-6, CRP, and Hb levels, suggesting that both surgical approaches exhibit comparable levels of invasiveness.

Two systematic reviews (Ang et al., 2023; Wang et al., 2024) indicate that there is no statistically significant difference in the incidence of complications such as dislocation, periprosthetic fracture, or venous thromboembolism between the two surgical approaches, which aligns with the findings of the current study. However, existing literature suggests that the DAA for THA in obese patients (BMI >30) is associated with a heightened risk of wound complications and periprosthetic joint infections when compared to the PLA (Shah et al., 2022). Consequently, it is advisable to utilize the DAA for patients with lower BMI and simpler anatomical configurations, while reserving the PLA for those with higher BMI and more complex anatomical structures that necessitate extensive surgical exposure.

The AI-HIP preoperative planning system used in this study can improve the efficiency and accuracy of THA preoperative planning compared with human manual planning, and the AI-HIP system adopts the unique Transformer_unet algorithm, which can realize the automatic and accurate segmentation of hip CT images in a short period of time to improve the clinical efficiency, and it has a high utility and clinical application value (Zhu et al., 2025). Previous studies have shown (Wu et al., 2020) that the complete compliance rate of acetabular-side and femoral-side prostheses in the AI-HIP group was 90.0% (27/30) and 83.3% (25/30), respectively, and in the manual preoperative planning group, it was 56.7% (17/30) and 53.3% (16/30); the difference between the two groups was statistically significant (P < 0.05), and the time-consuming preoperative planning in the AI-HIP group (5.02 ± 1.22) min, which was significantly reduced compared with (8.29 ± 2.08) min in the manual preoperative planning group, and the difference was statistically significant (t = −7.431, P < 0.05). Compared with traditional 2D and 3D preoperative planning, preoperative planning using the AI-HIP system resulted in less variability in measurement angles, more accurate prosthesis models, and easier operation, thus improving surgical outcomes (Zhu et al., 2025; Chen et al., 2022). Currently, AI preoperative planning is becoming more common in other joint areas in addition to its use in hip revision, and the integration of artificial intelligence (AI) into decision support systems for the diagnosis and treatment of orthopaedic diseases is one of the main directions in the development of orthopaedic technology (Andriollo et al., 2024). In knee surgery, AI preoperative planning is also widely used, and studies have shown (Lan et al., 2024) that in total knee arthroplasty the accuracy of prosthesis size prediction in the AI group was significantly higher than that in the 2D group, with the complete compliance rates of femoral and tibial prostheses in the AI reconstruction group of 90% (27/30) and 86.7% (26/30), respectively. The corresponding rates in the 2D template group were 66.7% (20/30) and 60% (18/30), with high prediction accuracy.

This study does have several limitations. Firstly, it primarily focused on early postoperative imaging and efficacy indices, lacking a follow-up investigation into long-term imaging outcomes, clinical results, and associated complications, which necessitates extended follow-up to enhance the comparative analysis. Secondly, the angles of FNA, AA, and CA were measured using Mimics and other related software for three-dimensional reconstruction. This process required manual separation of joint structures to eliminate metal artifacts and impurities, which may not have been entirely effective, leading to potential measurement errors. Thirdly, the patients were discharged with a comprehensive management platform for care, rehabilitation, and follow-up, which necessitated the use of iPhones. For older, less educated, and economically disadvantaged patients, follow-up and rehabilitation guidance were conducted via telephone, potentially introducing bias. Fourthly, other functional outcomes, such as hip mobility and gait analysis, were not evaluated in this study. Fifthly, functional outcome indicators, including pain function post-THA, were not correlated with the pairwise line and the AA, FNA, and CA measurements, and a multifactorial logistic regression analysis to assess the influence of various factors on postoperative outcomes was not performed. Future research could enhance the analysis of these influencing factors. Additionally, the AIHIP system, like other artificial intelligence tools, is not immune to errors (e.g., AI hallucinations) in its output, necessitating that the surgeon make judgments based on the actual intraoperative context. Finally, while the AI-HIP system was employed for preoperative planning in this study, it did not facilitate the selection of the surgical access route based on the patient’s baseline characteristics. Future developments could include intelligent predictive models that recommend surgical access routes tailored to the patient’s BMI, age, and underlying health conditions.

## 6 Conclusion

Both the DAA and the PLA, when utilized in conjunction with AI-HIP preoperative planning, can yield improved surgical outcomes. Specifically, DAA-THA demonstrates functional outcomes and anatomical alignment that are more closely aligned with AI-HIP assisted preoperative planning compared to PLA. Furthermore, DAA exhibits advantages over PLA in terms of postoperative pain, duration of hospitalization, functional scores during early follow-up, and scar length. However, no significant differences were observed between the two approaches regarding invasiveness and postoperative complications. Both techniques resulted in favorable postoperative follow-up outcomes and high levels of surgical satisfaction. Based on the findings of this study, the authors advocate for the preference of DAA as the surgical approach for THA, while also emphasizing the importance of considering patient-specific factors, as well as the surgeon’s preferences and experience.

## Data Availability

The raw data supporting the conclusions of this article will be made available by the authors, without undue reservation. Requests for data access can be directed to the corresponding author via email at z15500528876@163.com
